# Biomechanical Effects of a New Macrogeometry Design of Dental Implants: An In Vitro Experimental Analysis

**DOI:** 10.3390/jfb10040047

**Published:** 2019-10-25

**Authors:** Sergio Alexandre Gehrke, Leticia Pérez-Díaz, Patricia Mazón, Piedad N. De Aza

**Affiliations:** 1 Department of Research, Biotecnos, Cuareim 1483, Montevideo CP 11100, Uruguay; 2 Department of Bioengineering, Instituto de Bioingenieria, Universidad Miguel Hernández, Avda. Ferrocarril s/n, 03202 Elche (Alicante), Spain; piedad@umh.es; 3 Laboratorio de Interacciones Molecular, Facultad de Ciencias, Universidad de la Republica, Calle Iguá 4225, Montevideo 11400, Uruguay; letperez@gmail.com; 4 Departamento de Materiales, Óptica Tecnologia Electrónica, Universidad Miguel Hernández, Avda. Ferrocarril s/n, 03202 Elche (Alicante), Spain; pmazon@umh.es

**Keywords:** bone density, dental implants, healing chambers, initial stability, insertion torque, new implant macrogeometry

## Abstract

The purpose of the present study was to measure and compare the insertion torque, removal torque, and the implant stability quotient by resonance frequency analysis in different polyurethane block densities of two implant macrogeometries. Four different polyurethane synthetic bone blocks were used with three cortical thickness: Bone 1 with a cortical thickness of 1 mm, Bone 2 with a cortical thickness of 2 mm, Bone 3 with a cortical thickness of 3 mm, and Bone 4, which was totally cortical. Four groups were created in accordance with the implant macrogeometry (n = 10 per group) and surface treatment: G1—regular implant design without surface treatment; G2—regular implant design with surface treatment; G3—new implant design without surface treatment; G4—new implant design with surface treatment. All implants used were 4 mm in diameter and 10 mm in length and manufactured in commercially pure titanium (grade IV) by Implacil De Bortoli (São Paulo, Brazil). The implants were installed using a computed torque machine, and following installation of the implant, the stability quotient (implant stability quotient, ISQ) values were measured in two directions using Osstell devices. The data were analyzed by considering the 5% level of significance. All implant groups showed similar mean ISQ values without statistical differences (*p* > 0.05), for the same synthetic bone block: for Bone 1, the value was 57.7 ± 3.0; for Bone 2, it was 58.6 ± 2.2; for Bone 3, it was 60.6 ± 2.3; and for Bone 4, it was 68.5 ± 2.8. However, the insertion torque showed similar higher values for the regular macrogeometry (G1 and G2 groups) in comparison with the new implant macrogeometry (G3 and G4 groups). The analysis of the results found that primary stability does not simply depend on the insertion torque but also on the bone quality. In comparison with the regular implant macrogeometry, the new implant macrogeometry decreased the insertion torque without affecting the implant stability quotient values.

## 1. Introduction

The initial implant stability is a fundamental requisite to obtain osseointegration [1,2]. The main parameters that are involved are the bone condition (quality and quantity), the implant macrogeometry (design of the body and surface roughness), the osteotomy design, and the precise fit in the bone (friction coefficient) [3]. Thus, to achieve adequate osseointegration of the implant, it is of fundamental importance that a good primary stability of the implant is achieved after its installation into the bed prepared in the bone tissue. This is crucial for the long-term success of the implant [4,5]. 

The force for insertion of the implant into the bone tissue is related to the quality of the bone (density) and to the osteotomy performed (orifice size), generating compressive stresses at this contact interface between bone tissue and implant [6]. These obtained levels of compression determine the initial stability of the implant; sufficiently high values result in local ischemia of the bone and necrosis at the implant–tissue interface [7,8,9]. In this sense, several studies have proposed that approaching the diameter of the drilling (during the osteotomy) with the diameter of the implant that will be inserted into the bone can facilitate and improve osseointegration through a decrease in the bone compression [10,11]. Jimbo et al. (2014) showed, in a study using a dog model, that in the implants placed with high torque, the samples presented a certain amount of necrotic bone inside the implants threads, whereas in the samples where a larger amount of drilling was used, the samples presented substantial formation of new bone [11]. The free space created inside the implant threads, resulting from the drill–implant diameter ratio, was called healing chambers. Obviously, that procedure to create this healing chambers (over-drilling protocol) generate a sensible decrease of the final insertion torque level in the implant.

A low initial stability may allow micromovement of the implant during the healing period, and fibrous tissue may form at the interface between the bone and the implant and lead to failure [12]. However, when the implants have good primary stability values, the healing time may be shorter, as when the implants present low values of primary stability, they require longer waiting times to obtain adequate bone healing and consequent secondary stability [13]. This acquired information about the stability of the implants can help in determining the waiting time to obtain the healing of bone tissue around the implant for each case and in an individualized way, increasing the safety of the treatments, the effectiveness, and, in some cases, decreasing the time taken to complete the treatment [14].

In this sense, a new macrogeometry was developed with these concepts and the idea of “no bone compression” during the implant insertion without the loss of initial stability after implant installation. Healing chambers for the bone decompression were created in the implant thread body, generating spaces to deposit the bone during the implant insertion. However, the concept of higher insertion torque (IT), which translates into greater primary stability, cannot always be applied because bone quantity and quality vary significantly between patients [13].

Continuous monitoring in an objective and quantitative manner is important to determine the status of implant stability. Historically, the gold standard method used to evaluate the degree of osseointegration was microscopic or histologic analysis. However, due to the invasiveness of this method and related ethical issues, various other methods of analysis have been proposed: Radiographs, cutting torque resistance, reverse torque, modal analysis, and resonance frequency analysis [15,16]. Thus, the purpose of the present study was to measure and compare the insertion torque, removal torque, and the implant stability quotient by resonance frequency analysis in different polyurethane block densities of two implant macrogeometries. Moreover, we analyze the effect of the threads passage of both implant models during their insertion and after removed into the polyurethane block totally cortical.

## 2. Materials and Methods

### 2.1. Synthetic Bone Characteristics

**Figure 1:**
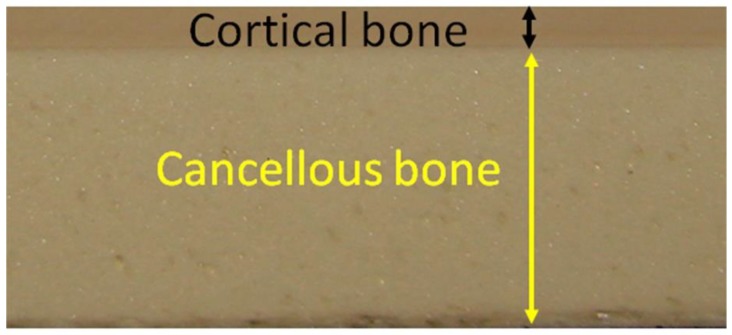
Representative image of the synthetic bone blocks showing the cortical portion (black arrow line) and cancellous bone portion (yellow arrow line).

**Figure 2:**
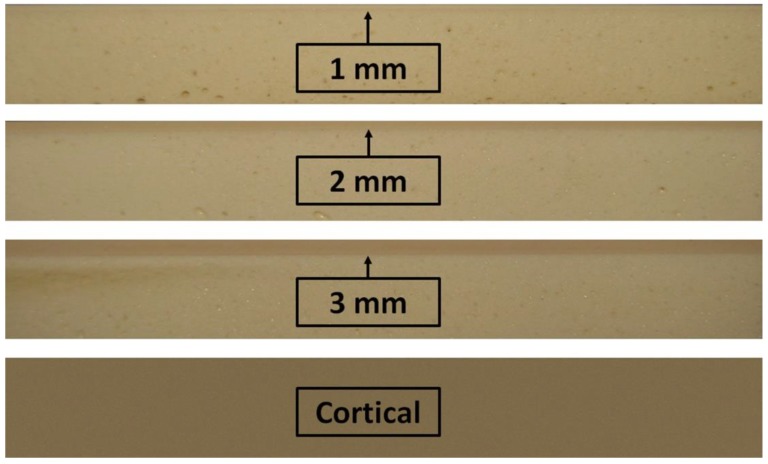
Image of the synthetic bone blocks with three cortical thicknesses (1, 2, and 3 mm) and a totally cortical bone block.

Synthetic bone blocks of polyurethane (Nacional Ossos, São Paulo, Brazil) with cortical and medullar portions were used. The cortical portion was fabricated in a density of 40 pounds per cubic foot (PCF) or 0.64 g/cm^3^, and the cancellous bone portion of all blocks presented a density of 15 PCF or 0.24 g/cm^3^ (Figure 1). In humans, the mean bone mineral density of the posterior maxilla is 0.31 g/cm^3^ and that of the anterior maxilla is 0.55 g/cm^3^ [17]. The block configurations used presented a height of 2 cm, a width of 2 cm, a length of 13 cm, and four different cortical thicknesses at 1, 2, and 3 mm and totally cortical (Figure 2). Polyurethane blocks were used at different densities to simulate bone in an in vitro setting. Polyurethane is considered to be the standard material for performing mechanical tests on orthopedic implants [18,19,20,21].

### 2.2. Implant Characteristics and Group Distribution

**Figure 3:**
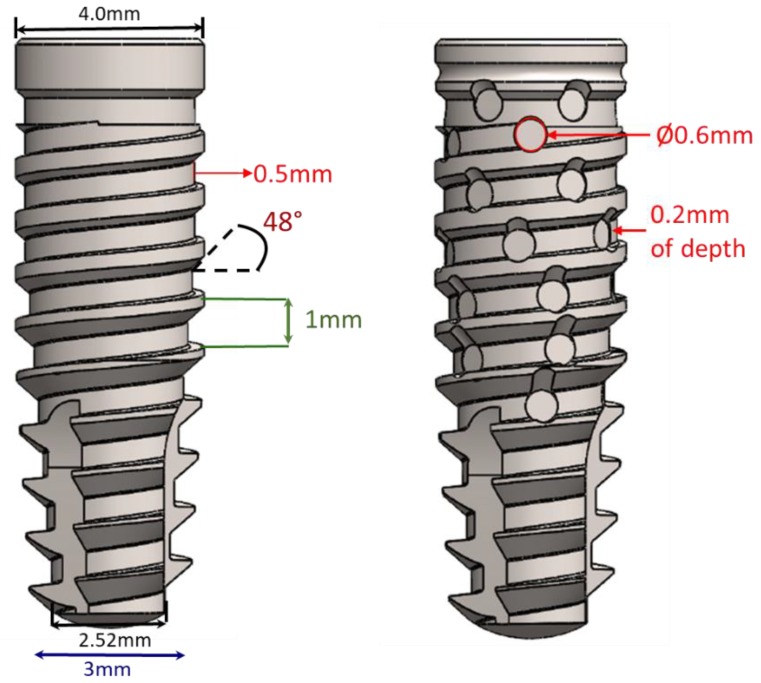
Schematic image of the implants and the dimensional details of the regular and new macrogeometry, respectively.

The conical regular design shows progressive trapezoidal threads, a cervical portion with 1 mm of plane configuration in the final cervical area, and a Morse taper connection, whereas the new conical implant design shows progressive trapezoidal threads, a cervical portion with 1 mm of plane configuration in the final cervical area, healing chambers in the threads, and a Morse taper connection. Figure 3 show a schematic image of both implant designs.

Both models were tested with a surface treated with a blasting process plus acid conditioning and an untreated (machined) surface. The surface-treated implants were blasted with 50 to 100 µm of TiO_2_ microparticles, cleaned ultrasonically with alkaline solution, washed in distilled water, and conditioned with maleic acid (HO_2_CCHCHCO_2_H). After these treatments, three implants from each group were used to determine the roughness parameters using scanning electron microscopy (SEM) and atomic force microscopy (AFM). The surface morphology of the samples in both groups was examined under SEM (JEOL, model JSM 6490-LV, Tokyo, Japan) using the secondary electron (SE) detection mode with an acceleration of 20 kV and a spot size of 4.0. For a direct comparison of the surface morphology, the same magnification (1000×) was selected for all samples. Then, the samples were used to generate a series of 3D images using a scanning probe microscope (AFM) (Bruker, Santa Barbara, CA, USA). To measure the surface roughness parameters, an optical laser profilometer (Perthometer S2, Mahr GmbH, Göttingen, Germany) was used, where Ra is the absolute value of all profile points, and Rz is the value of the absolute heights of the five highest peaks and the depths of the five deepest valleys. 

Four groups (n = 10 per group) were formed according to the implant design (Figure 2): Group 1 (G1)—regular conical design without surface treatment; group 2 (G2)—regular conical design with surface treatment; group 3 (G3)—new conical design without surface treatment; and group 4 (G4)—new conical design with surface treatment. The dimension of all implants used were 4 mm in diameter and 10 mm in length. The implants were manufactured by Implacil De Bortoli (São Paulo, Brazil).

### 2.3. Implant Management and Biomechanical Analysis

**Figure 4:**
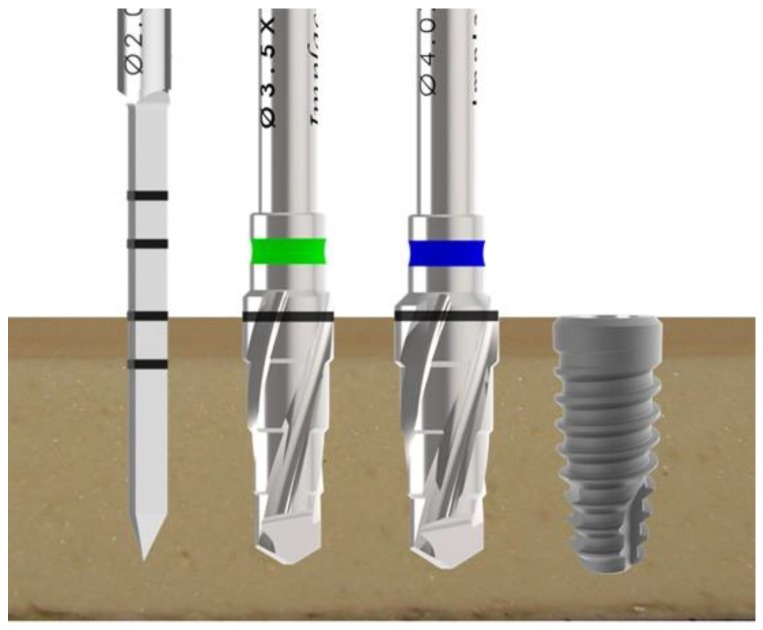
Schematic image of the drilling sequence used to install the implants of all groups.

The drilling was done in accordance with the manufacturer’s designation for each implant model. All osteotomies were prepared using a bench drill with 20 N of force using a surgical drill at a rotational speed of 1200 rpm under intense external irrigation with saline solution, using a predeterminate drilling sequence of the implant system (Figure 4): initially a Ø2 mm drill, Ø3.5 mm conical drill, and Ø4.0 mm conical drill. 

**Figure 5:**
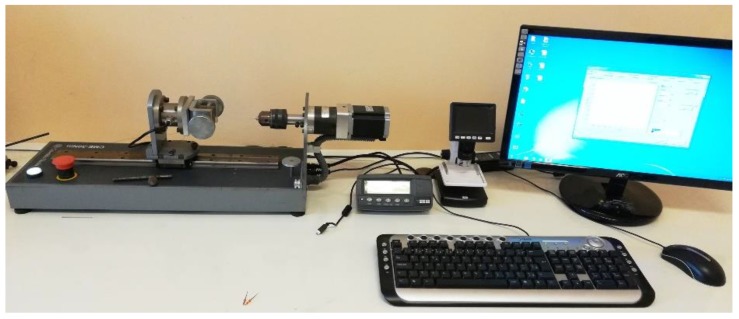
Image of the torque machine used to install and remove the implants into the synthetic bone blocks.

The implant installation was done using a computed torquimeter machine (Torque BioPDI, São Paulo, Brazil), as shown in Figure 5. 

All implants were installed at the bone level. The maximal insertion torque value was recorded for each sample, and then the implants were removed, and the maximal removal torque was recorded. To analyze and compare the viscoelastic properties of each bone model, an equation (the rule of three) was used which used these data (insertion and removal torque values) to calculate the torque reduction (TR) as a percentage:

(1) $$\mathrm{Torque}\;\mathrm{reduction}=\frac{\mathrm{insertion}\;\mathrm{torque}}{\mathrm{removal}\;\mathrm{torque}}=\frac{100\%}{X}$$

**Figure 6:**
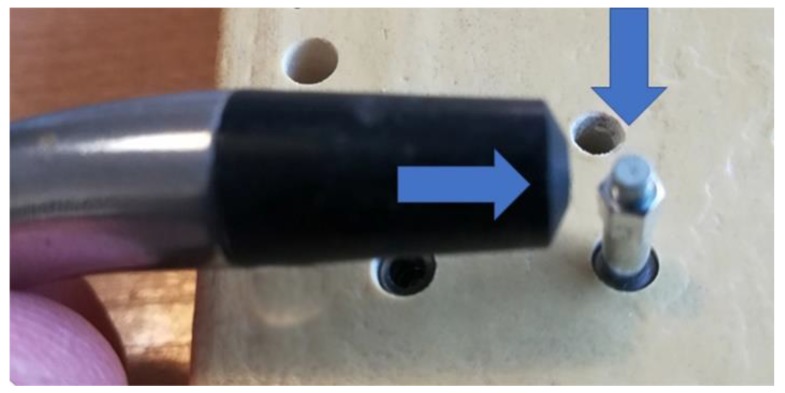
Representative image showing the two directions used to measure the implant stability quotient (ISQ) using the Osstell device.

Following each installation, the implant stability quotient (ISQ) was measured using the Osstell Mentor device (Osstell, Göteborg, Sweden). The smart peg was screwed in the implant, and a torque of 10 N·cm was applied [22]. The ISQ values were represented on a scale from 1 to 100. The measurement was performed in 2 directions for each sample (Figure 6), and an average value was determined for each implant. 

### 2.4. Analysis of the Effect of the Threads Passage

The effect promoted by the threads passage in the polyurethane block of both implant models (regular and new macrogeometry) was evaluated and described using photographic images obtained after the implant insertion and removal in a fully cortical block model (Bone 4). This model was selected, since it is among all, which is more evident the effect (scars) promoted during the implant passage.

### 2.5. Statistical Analysis

The IT and ISQ values were summarized using means and standard deviations. One-way analysis of variance was used to compare the mean IT and ISQ values. The Shapiro–Wilk test was used to test the normality. The Pearson’s correlation coefficient was used to evaluate the correlation between the IT and the ISQ at implant placement. All analyses were done using GraphPad Prism version 5.01 for Windows (GraphPad Software, San Diego, CA, USA). When the *p*-value was <0.05, the differences were considered significant.

## 3. Results

**Figure 7:**
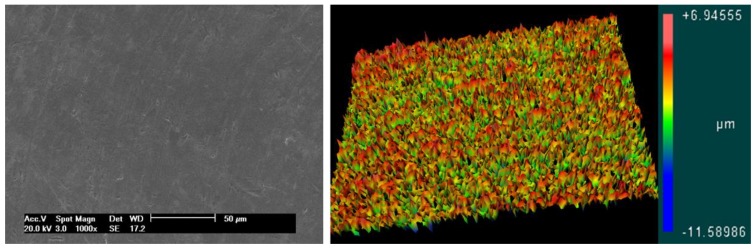
Representative image of the smooth surface obtained by scanning electron microscopy (SEM) and atomic force microscopy (AFM), respectively.

**Figure 8:**
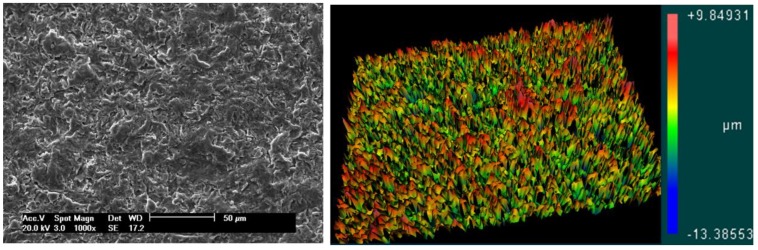
Representative image of the rough surface obtained by SEM and AFM, respectively.

No morphological and roughness differences were observed between samples from groups G1 and G3 and between groups G2 and G4. The images shown in Figure 7 represent the smooth surface groups (G1 and G3 groups), and the images in Figure 8 represent the rough surface groups (G2 and G4 groups). The profilometer analysis showed the means and standard deviations of the absolute values of all profile points (Ra): 0.11 ± 0.05 μm for the smooth surface implants and 0.85 ± 0.13 μm for the rough surface implants. The root-mean-square of the values of all points (Rq) was 0.22 ± 0.09 μm for the smooth surface implants and 1.14 ± 0.09 μm for the rough surface implants, and the average value of the absolute heights of the five highest peaks and the depths of the five deepest valleys (Rz) was 1.12 ± 0.18 μm for the smooth surface implants and 5.11 ± 0.54 μm for the rough surface implants.

**Figure 9:**
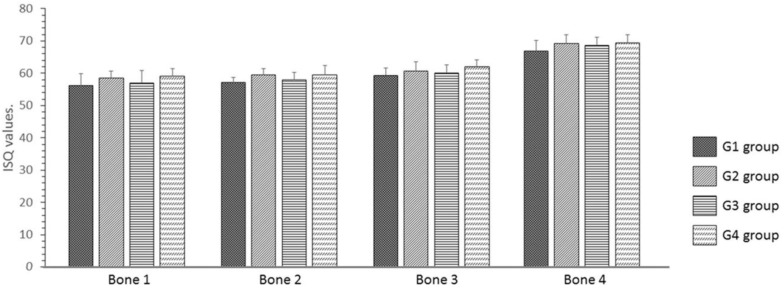
Bar graph of the distribution of ISQ values for each model of synthetic bone block in each group proposed.

The ISQ values showed similar values for both implant macrogeometries with the same treatment. However, the G2 and G4 groups, in which the implants received surface treatment (rough surface), showed values slightly higher than those of the G1 and G3 groups (without treatment on the implant surface) in all bone models. The data values (mean and standard deviation) and statistical comparison are summarized in Table 1 and demonstrated in the line graph shown in Figure 9. 

The torque caused by the insertion and removal of the implants presented similar values for the same macrogeometry; however, different values were shown between the two macrogeometries, where the regular macrogeometry showed superior values (a mean of ~17% higher) for both groups (G1 and G2 group) in comparison with the G3 and G4 groups, independent of the bone density. The data values (mean and standard deviation) are summarized in Table 2.

**Figure 10:**
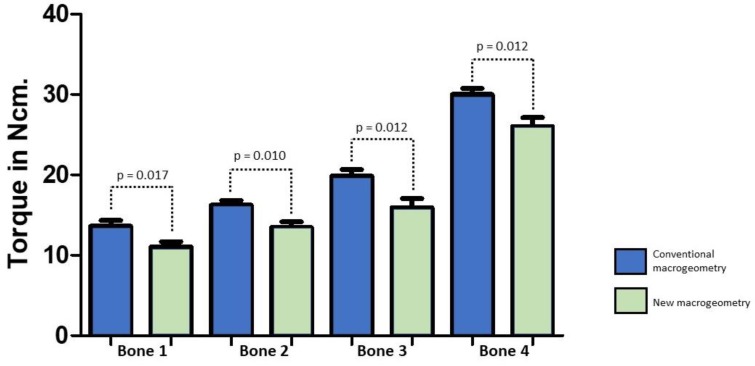
Bar graph of the distribution of insertion torque values and statistical analysis for the two implant macrogeometries in each synthetic bone block model.

The insertion torque values obtained from the groups with the same macrogeometry were compared statistically to determine possible differences (G1 and G2 versus G3 and G4) considering that the surface treatment did not change the torque values in this test for the samples evaluated in our study. The bar graph in Figure 10 show the comparative values of the insertion torques of the two macrogeometries tested and the statistical analysis, which showed statistical differences between both implant macrogeometries (*p* < 0.05).

Regarding the overall mean of the bone models, the values of removal torque were 44.6% smaller than the insertion torque for the groups with regular macrogeometry (G1 and G2 groups) and 39.4% for the groups with the new implant macrogeometry (G3 and G4 groups). The data collected on the removal torque of all bone models and groups are summarized in Table 3.

Overall, the calculated reduction torque was 50% for the G1 and G2 groups and 39% for G3 and G4 groups in Bone 1; for the Bone 2, the reduction torque was 53% in the G1 and G2 groups and 45% in the G3 and G4; for the Bone 3, it was 55% for the G1 and G2 groups and 47% for the G3 and G4 groups; and for the Bone 4, the mean value was 32% for all groups.

No correlation was detected between the insertion torque and stability (ISQ) values for the groups. The correlation analysis between the insertion torque and initial stability quotient is shown in Table 4.

**Figure 11:**
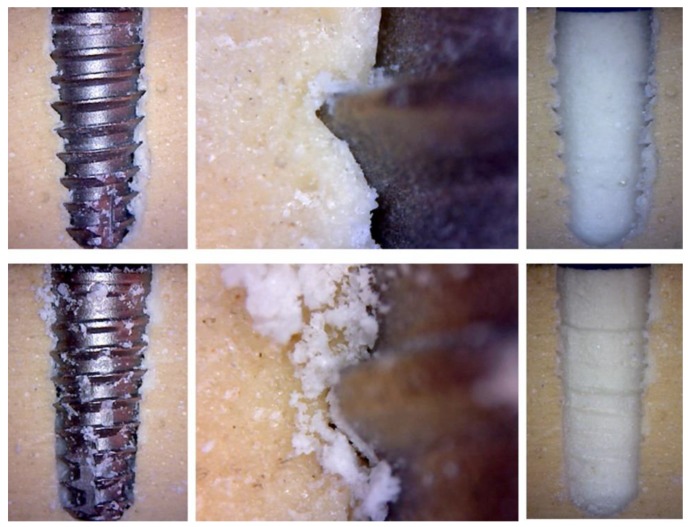
Representative image of the effect (scars promoted) of threads passage of the two implant macrogeometries used. The atop image sequence shows the regular macrogeometry, and the bottom image sequence shows the new macrogeometry during the implant insertion and after the implant removal.

By analyzing the effect of the threads passage of both implant models during their insertion into the polyurethane blocks, it was possible to observe that the samples with regular macrogeometry (groups G1 and G2) promoted a few cuts of the bone during the passage of the threads, while in the model with the new macrogeometry, we observed that the bone tissue was cut and carried by the threads, as shown in the images of Figure 11. Moreover, the quantity of bone particles that was observed to be deposited on the surface of the new macrogeometry was higher than that on the regular macrogeometry. After removing the implant, we observed that the bone of the new macrogeometry site showed a greater marking produced by the passage of the threads in comparison with the regular macrogeometry.

## 4. Discussion

The present study evaluated the insertion and removal torque of two implants with different macrogeometries (regular and a new design), with and without surface treatment. We also measured the primary stability through a resonance frequency analysis in different bone densities. The primary stability of the implants during installation was determined by the bone quality, bone quantity, implant geometry, and installation technique. Several authors have shown the importance of primary stability in obtaining osseointegration of dental implants [23,24,25], and failure to obtain efficient primary stability can lead to early implant loss [26]. In addition to these factors, it has been demonstrated in other studies that treatment of the implant surface can influence the results of primary stability [23,24,25]. 

The implants of groups G3 and G4 with the new macrogeometry were developed to improve and accelerate osseointegration based on the hypothesis that no bone compression would occur during installation [27]. This concept has been demonstrated in several recent studies [11,27], which tested an undersized osteotomy to decrease the bone compression during the implant insertion. The histological results showed that the maneuver improves and accelerates the osseointegration of the implants. However, the authors stated that the technique can promote a decrease in the initial stability of the implants. In this sense, the idea of a new macrogeometry with healing chambers incorporated into the implant body does not alter the size of the osteotomy but generates spaces to make the bone decompress. Then, we proposed a comparison of this new macrogeometry with a regular macrogeometry to evaluate the relationship between less bone compression during the implant installation with the obtention of the insertion torque and the initial stability. The results show that, in comparison with the regular macrogeometry, the insertion torque of the new macrogeometry was less than 16% (overall of the mean), while the initial stability was not affected. 

In all groups the torque removal was significatively less than the insertion torque. However, the implant groups with the new macrogeometry (G3 and G4 groups) showed a smaller reduction torque change than the implant groups with the regular macrogeometry (G1 and G2 groups), except for the fully cortical bone model (Bone 4), where the reduction torque was equal for both tested implant macrogeometries. The lower values of reduction torque suggest that the presence of decompression chambers decreased the stresses over the bone where the implants were inserted. According to Ahn et al. [28], the difference between insertion torque and removal torque is due to the restricted viscoelastic properties of the surrounding artificial bone, which results in less resistance during removal. Bone 3 (with a cortical thickness of 3 mm) showed a higher reduction in torque, which presented more viscosity in relation to the other bone models tested, whereas Bone 4 showed less viscosity.

Regarding the synthetic bone blocks used for in vitro analysis, the rigid polyurethane foam with homogeneous and good characteristics is considered an ideal material and is in accordance with the ASTM standard F1839-08 (1997) [29]. Thus, we used a polyurethane foam density of 0.48 g/cm³ in the cortical portion, considering that the mean of cortical bone density in human maxilla is 0.31 g/cm³ for the posterior area and 0.45 g/cm³ for the anterior area [29]. The densities of polyurethane foam used in the present study was of 15 pounds per cubic foot (pcf), corresponding to a density of 0.24 g/cm^3^ (similar to the D3 bone type by Mish [30,31]); and 40 pcf, corresponding to 0.55 g/cm^3^ (similar to D1 bone by Mish [30,31]). Cancellous bone receives and dissipates the forces generated by mastication after implant osseointegration more efficiently; however, to obtain the initial stability, cortical bone is more important because it has high density and resistance (~40% more) in comparison to medullary bone [10]. 

Resonance frequency analysis (RFA) measurement using Osstell Mentor is frequently used to evaluate the implant stability in preclinical and clinical studies [32,33,34]. This technique has been widely used because it is not invasive and does not require extra procedures to obtain the data. However, this method revealed the absence of mobility of the installed implant and not the bone quantity at the implant–bone interface [35,36]. The determination of good osseointegration is directly related to the absence of movement at the bone–implant interface in the different types of bone density [31]. Therefore, the lack of micromovement determined by a rigid primary stability and healing period free from external stimuli is originally a prerequisite for obtaining a satisfactory clinical result [37]. However, for implants placed in low density bone, the stability indices (RFA) at the end of the osseointegration process will be similar to those of medium- and high-density bone implants [38]. Different from this result, we did not find a correlation between the values of the RFA at the moment of implant installation and the torque in the fixation of the implants, which was also reported in another study [39]. These results indicate that we must use greater caution when conferring the analysis of frequency of dental implants, because the limits of height and width of the implants, as well as factors of bone density, can influence its result.

The results showed that the insertion torque increased in accordance to the bone density increase, whereas the implant stability (ISQ) showed no variation in Bone 1–3 models. The results found in our study showed that there is no correlation between the two parameters tested. Therefore, the only factors that showed a positive correlation between the IT and the ISQ value were the bone density and thickness of the cortical bone. This result is in accordance with other publications [12,40]. Moreover, Lages et al. reported that the clinician should choose only one of the methods to determine the primary stability of implants, as these are independent and incomparable methods [12].

The interaction between the implant and the adjacent bone immediately after its insertion depends mainly on the macrogeometry of the implant and the topography of its surface [41,42]. However, some studies in the literature still question the influence of surface treatment on the primary stability [12,43,44,45,46] corroborated the results obtained in the present study, where the two implant designs did not present a statistical difference in the insertion torque values between the treated and non-treated surfaces.

Several authors evaluated the strength and stiffness of the shear bone–implant interface through resonance frequency analysis to search for information about the degree of contact in this interface [2,3,6,47,48]. In the present study, when evaluating the initial stability of the implants inserted in the synthetic cortical bone (40 PCF), it was verified that all implants obtained the highest values. However, the implants with regular macrogeometry showed superior values due to having greater contact and friction surface between the screw and the material [43,49]. 

In the resonance frequency analysis, larger values were observed in implants that underwent surface treatment (G2 and G3 groups) compared to the machined ones (G1 and G3 groups), corroborating findings described by other authors [50]. Despite these data, the presence of surface treatment was not associated with a significant difference between machined and treated implants on all substrates. These results corroborate with studies in the literature [43,51] and suggest that RFA is not sensitive enough to detect minor alterations, such as the surface treatment of the implants.

Some studies have shown that, due to lateral cortical compression of low quality bone sites, conical implants have a higher IT than cylindrical implants [52,53,54,55]. These studies show that the conical implants present higher IT values when compared to cylindrical ones when inserted in the swine bone and artificial polyurethane bone of 15 PCF, suggesting that the use of this type of screw in low density bones is appropriate [53]. The conical implant design was selected and used in the present study because it presents a higher insertion torque when compared to a cylindrical implant design, as shown in other studies [26,53,54,55,56].

The absence of correlation between RFA and IT has been reported in several studies [39,41,57]. However, an important finding was the reduction in the insertion torque values found, especially in the groups of the regular implant macrogeometry (G1 and G2), which can be considered as a loss of primary stability, even in the presence of high ISQ values. As previously described, the higher bone density values generate a greater the possibility of reducing the initial torque measured by the viscosity of this tissue. Certainly, new studies that evaluate and corroborate these findings should be performed.

Regarding the limitations of the present study, we can report that only the mechanical aspects of the effect of surface format and treatment were evaluated, that is, biological factors such as bone response, individual characteristics, local variations in human bone, and the surgical technique, which also influence primary stability in a clinical situation. Regarding the material (synthetic bone blocks) used, inhomogeneity due to the presence of fat, bone marrow, and blood inside the real human bone is challenging to model in a foam model. It was assumed that the contributions of these components are negligible. The current results apply to the implants of dimensions as described. Hence, caution should be exercised while extrapolating the results to other implant types. However, it has been demonstrated that the surface treatment, shape, and difference in implant threads depends on the correlation between shape and bone density in order to promote an optimal biomechanical condition for osseointegration. Another important observation is that, in our study, the foam was destroyed by the threads. However, we compared implants on the basis that the bone would be damaged in the same way as foam. Therefore, it is important to note that a bone of the same density may be much more or less resistant to the cut, and the result of the thread crossing may be completely different. 

## 5. Conclusions

Within the limitations of the present in vitro study, it can be concluded that, in comparison with the regular implant macrogeometry, the new implant macrogeometry presented low insertion torque values without affecting the implant stability quotient (ISQ) values. In addition, the insertion torque and ISQ values did not differ in relation to the surface treatment of the tested implants. Finally, no correlation was found between the insertion torque and ISQ values measured by the Osstell device.

## Figures and Tables

**Table 1:** Mean, standard deviation, and statistical analysis of the measured values of ISQ for each group in the different synthetic bone blocks.

Parameter	Group G1	Group G2	Group G3	Group G4	*p*-Value
Bone 1	56.2 ± 3.71	58.5 ± 2.17	57.0 ± 3.79	59.2 ± 2.32	0.4093
Bone 2	57.2 ± 1.60	59.5 ± 1.87	58.0 ± 2.28	59.5 ± 2.88	0.4842
Bone 3	59.3 ± 2.34	60.7 ± 2.17	60.2 ± 2.48	62.0 ± 2.10	0.2105
Bone 4	66.8 ± 3.25	69.2 ± 2.79	68.7 ± 2.42	69.3 ± 2.66	0.2551

**Table 2:** Means and standard deviations of the measured values of insertion torque for each group in the different synthetic bone blocks.

Parameter	Group G1	Group G2	Group G3	Group G4
Bone 1	13.4 ± 2.04	13.8 ± 2.55	11.0 ± 2.06	11.2 ± 2.19
Bone 2	16.0 ± 1.93	16.4 ± 1.98	13.1 ± 2.58	13.8 ± 2.03
Bone 3	19.5 ± 2.39	20.2 ± 2.89	15.7 ± 3.99	16.2 ± 3.36
Bone 4	29.6 ± 2.33	30.4 ± 2.85	25.8 ± 3.89	26.2 ± 3.32

**Table 3:** Means and standard deviations of the measured values of removal torque for each group in the different synthetic bone blocks.

Parameter	Group G1	Group G2	Group G3	Group G4
Bone 1	6.72 ± 1.43	6.92 ± 1.44	6.50 ± 1.52	6.94 ± 1.32
Bone 2	7.46 ± 1.52	7.84 ± 1.46	7.10 ± 1.57	7.48 ± 1.54
Bone 3	8.46 ± 1.50	9.78 ± 1.70	8.34 ± 1.97	8.58 ± 1.72
Bone 4	20.22 ± 1.86	20.78 ± 2.05	17.14 ± 1.85	18.38 ± 1.40

**Table 4:** Pearson correlation analysis and *p* values of the insertion torque and initial stability quotient of all groups in all bone models.

Group	Bone 1	Bone 2	Bone 3	Bone 4
G1	r = 0.334/*p* = 0.497	r = −0.395/*p* = 0.419	r = −0.348/*p* = 0.497	r = −0.486/*p* = 0.053
G2	r = 0.358/*p* = 0.497	r = 0.086/*p* = 0.919	r = −0.717/*p* = 0.136	r = −0.152/*p* = 0.803
G3	r = 0.029/*p* = 1.000	r = −0.541/*p* = 0.058	r = −0.429/*p* = 0.419	r = 0.435/*p* = 0.419
G4	r = 0.086/*p* = 0.919	r = 0.486/*p* = 0.356	r = −0.058/*p* = 0.919	r = −0.395/*p* = 0.419

r = correlation coefficient; *p* = value of the statistical difference.
